# Factors associated with medication amounts considered excessive among university students: a questionnaire survey of pharmacy students and those in non-medical schools

**DOI:** 10.1186/s12913-017-2431-9

**Published:** 2017-07-11

**Authors:** Megumi Saito, Noriko Ando-Tanabe, Etsuko Arita

**Affiliations:** Department of Medical Psychology, Pharmaceutical Education Center, Kitasato University School of Pharmacy, 5-9-1, Shirokane, Minato-ku, Tokyo, 108-8641 Japan

**Keywords:** Attitude toward medicine dosage, Personalized service, Medication

## Abstract

**Background:**

Better insight and knowledge on factors associated with perception of medication numbers and amounts would contribute greatly to our current understanding of patient psychological response regarding taking medications, and would allow us to improve drug administration support and adherence. This study explored associations between attitudes toward medication dosage in a questionnaire survey that examined demographic characteristics, the number of tablets and types of prescription medications considered excessive by participants, current medication and supplement use, personal experiences with medications, and perceptions surrounding medications.

**Methods:**

An original anonymous questionnaire was used for this survey. A total of 934 university students completed and returned surveys with no missing data.

**Results:**

Mean values ± standard deviation for excessive thresholds for tablets and types of medications reported by all participants were 4.21 ± 1.63 tablets and 4.00 ± 1.25 medications, respectively. The number of tablets considered excessive was analyzed using a multiple regression model, which accounted for the variance (model-adjusted *R* ^2^ = 0.095, *p* < 0.001) between statistically significant factors, including personal experience with a major illness, supplement use, aversion to taking medications, gender, university departmental affiliation, and experience with family members or acquaintances who took excessive amounts of medications **(|**beta**| >** 0.094, *p* < 0.01). The number of medications considered excessive was subject to a multiple regression analysis (model-adjusted *R* ^2^ = 0.087 *p* < 0.01), which revealed statistically significant factors, including personal experience with a major illness, prescription medication use, aversion to taking medications, gender, university departmental affiliation, and experience with family members or acquaintances who took excessive amounts of medications (|beta| > 0.084, *p* < 0.01).

**Conclusions:**

Individual attitudes toward medication dosage are influenced by individual factors. Thus, patients should be provided with personalized advice when they receive medication instructions.

**Electronic supplementary material:**

The online version of this article (doi:10.1186/s12913-017-2431-9) contains supplementary material, which is available to authorized users.

## Background

For pharmacological treatments and medications to be effective, drug compliance and adherence are crucial. Unfortunately, compliance and adherence are low in some patients. According to the World Health Organization, factors associated with drug adherence among patients include 1) the manner in which health care providers interact and communicate with their patients; 2) health care delivery system-related factors, such as the accessibility of medical care services, support system for patient education, and regional support activities; and finally, 3) patient characteristics, such as patient condition, level of trust in the treatment, and other emotional factors [1]. Moreover, a large study of chronic disease patients in the United States recently revealed that “the financial hardship of paying for medication,” “fear or experience of side effects,” “generic concerns about medication,” and “lack of a perceived need for medication” were among the reasons for medication non-fulfillment and medication non-persistence [2].

One factor associated with the levels of drug compliance and adherence among patients is the number of pills and medications that are prescribed. Previous surveys that targeted patients revealed that one reason why patients forget or choose not to take a medication is that “there are so many drug types/amounts [3, 4].” Some patients reportedly hope to decrease the number of medications they take [5, 6]. One study queried patients receiving treatment for lifestyle diseases, and asked them if they would consider it beneficial if their prescribed number of types of medication decreased by even one. Of the patients taking the same number of medications, some responded that they strongly agreed, while some did not agree at all [5], revealing a high variability in patient perception of the number of medications.

Previous studies have suggested that drug dose affects adherence to medication. Thus, the drug dose that patients can tolerate at one time should be considered. To our knowledge, few studies have assessed the amount/type of drug that patients consider excessive to take at one time. Thus, we focused on the amount of drugs considered excessive by patients to take at one time (tablets and types of medication) and included an item pertaining to this in the questionnaire. We also investigated the influence of various individual factors on the amount of drugs considered excessive to take at one time. This study was in the pilot phase.

Better insight and knowledge on factors associated with perception of medication numbers and amounts would contribute greatly to our current understanding of patient psychological response regarding taking medications. This study aimed to examine factors which affected the number of medications considered excessive, as well as how these numbers vary. The questionnaire used in this study examined how personal background factors, perception of drugs, and general attitude about taking medications were associated with the number of tablets/types of medications which would be considered excessive for self-use by the participants themselves (hereafter, ‘excessive threshold’). In particular, the present study survey targeted college students. Individuals in their late teens to early 20s have less experience with disease and medication than do the elderly. That said, while their medication experience is somewhat limited, college students are potential candidates for medication, as they are embarking on stages in life when they might initiate routine medication use in the near future. This study focuses on potential candidates for medication rather than those actually taking medications, with the aim to clarify awareness of medication among a population that has not yet initiated medication use. The findings of this study should help to improve the introduction of and adherence to medication among those who are newly initiated to medication.

## Methods

### Participants

This study targeted 2nd-year, 4th-year, and 6th-year students of the Kitasato University School of Pharmacy (hereafter, pharmacy students) as well as students of Aoyama Gakuin University (hereafter, students in non-medical schools). Study subjects included 802 pharmacy students and an unknown number of students in non-medical schools.

### Questionnaire

The study questionnaire was originally developed by a pharmacy student (M.S.), and checked and revised by a researcher with a Ph.D. (N.T.). Some pharmacy students were asked to complete the revised questionnaire while noting whether the questionnaire was readable, understandable, subject to misinterpretation, or not. They provided feedback about how it might be improved, after which the final version of the questionnaire was developed by M.S., N.T., and E.A. (Ph.D.). The questionnaire survey comprised questions related to participant background information; items related to taking medications including history of side effects from medications and routine usage of medications and supplements; items related to participant perception of medications including the level of trust, sense of danger, and sense of aversion toward drugs; items related to attitudes and perception of taking medications; and the number of tablets/types of medications that would be considered excessive for self-administration in one sitting (‘excessive threshold’). Details on each item are shown in Tables 1, 2, 3, 4 and 5.

**Table 1:** Questionnaire items

Demographic characteristics	
Gender	
University (Pharmacy, Non-medical schools)	
Presence of roommates	
Allergy history	
History of major illness	
Items pertaining to medication/supplements	
History of side effects	
Routine usage of prescription medicine	
Routine usage of over-the-counter drugs	
Routine usage of supplements	
Emotional response to medication	
Trust medication	
Impression that medication is dangerous	
Feel disgust about taking medication	
Experience with, response to, and recognition of medication	
Take medicine instantly upon becoming ill	
Experience with taking “too many” medications	
Experience with family members/close acquaintances who take “too many” medications	
Reacts if a doctor prescribes “too many” medications	
Reacts if a doctor prescribes “too many” medications	
Understanding of drug efficacy	
Compliance with medication

**Table 2:** Participant background information and ‘excessive threshold’

		Mean (SD)		*t* value	Effect size	Observed power (1-β)
Gender [n (%)]		Male [333 (35.7)]	Female [601 (64.3)]			
	Tablets	4.47 (2.05)	4.07 (1.33)	3.168**	*d* = 0.25	0.96
	Types of medications	4.16 (1.49)	3.91 (1.09)	2.744**	*d* = 0.20	0.83
University [n (%)]		Pharmacy [562 (60.2)]	Non-medical schools [372 (39.8)]			
	Tablets	4.34 (1.71)	4.03 (1.49)	2.894**	*d* = 0.19	0.81
	Types of medications	4.13 (1.26)	3.79 (1.20)	4.171***	*d* = 0.27	0.98
Presence of cohabiters [n (%)]		Yes [727 (77.8)]	No [207 (22.2)]			
	Tablets	4.20 (1.57)	4.26 (1.83)	0.413	-	-
	Types of medications	4.03 (1.22)	3.88 (1.35)	1.567	-	-
Allergy history [n (%)]		Yes [172 (18.4)]	No [762 (81.6)]			
	Tablets	4.34 (1.79)	4.19 (1.59)	1.091	-	-
	Types of medications	3.99 (1.05)	4.00 (1.29)	0.034	-	-
History of major illness [n (%)]		Yes [175 (18.7)]	No [759 (81.3)]			
	Tablets	4.79 (2.42)	4.08 (1.35)	3.741***	*d* = 0.44	1.00
	Types of medications	4.35 (1.53)	3.92 (1.16)	3.537***	*d* = 0.35	0.99

*p < 0.05, **p < 0.01, ***p < 0.001

**Table 3:** History of side effects from medications, routine usage of medications/supplements, and ‘excessive threshold’

		Mean (SD)			*t* value/*F* value	Effect size	Observed power(1-β)
History of side effects [n (%)]		Yes [207 (22.2)]	No [727 (77.8)]				
	Tablets	4.33 (1.85)	4.18 (1.56)		1.461	-	-
	Types of medications	4.11 (1.34)	3.96 (1.22)		1.114	-	-
Routine usage of prescription medicine [n (%)]		Yes [163 (17.5)]	No [771 (82.5)]				
	Tablets	4.47 (1.66)	4.16 (1.62)		2.198*	*d* = 0.19	0.60
	Types of medications	4.31 (1.25)	3.93 (1.24)		3.542***	*d* = 0.31	0.95
Routine usage of over-the-counter drugs [n (%)]		Yes [23 (2.5)]	Only when unwell [608 (65.1)]	No [303 (32.4)]			
	Tablets	4.89 (2.02)	4.2 (1.44)	4.18 (1.93)	0.307	-	-
	Types of medications	4.02 (0.91)	4.02 (1.27)	3.95 (1.23)	2.051	-	-
Routine usage of supplements [n (%)]		Yes [124 (13.3)]	Only when unwell [125 (13.4)]	No [685 (73.3)]			
	Tablets	4.96 (2.63)^a, b^	4.17 (1.51)^a^	4.09 (1.36)^b^	6.583**	*f* = 0.18	1
	Types of medications	4.22 (1.26)^c^	4.15 (1.55)	3.93 (1.18)^c^	3.615*	*f* = 0.09	0.71

*p < 0.05, **p < 0.01, ***p < 0.001; ^a-c^indicates significant differences between parameters (*p* < 0.05)

^a^ *d* = 0.37, 1-β = 0.83; ^b^ *d* = 0.54, 1-β = 1.00; ^c^ *d* = 0.24, 1-β = 0.69 (*d*: effect size for each post-hoc analysis, 1-β: observed power)

**Table 4:** Level of trust, sense of danger, and sense of aversion toward drugs (including over-the-counter drugs and supplements) and ‘excessive threshold’

		Mean (SD)				*F* value	Effect size	Observed power (1-β)
Do you think medicine is reliable? [n (%)]		Extremely reliable [229 (24.5)]	Reliable [669 (71.6)]	Somewhat reliable [27 (2.9)]	Not reliable [9 (1.0)]^†^			
	Tablets	4.44 (1.82)	4.15 (1.55)	3.70 (1.14)	4.67 (2.87)	4.070*	*f* = 0.09	0.72
	Types of medications	4.19 (1.37)^a,b^	3.96 (1.20)^a^	3.46 (0.95)^b^	3.67 (1.50)	5.616**	*f* = 0.11	0.86
Do you think medicine is dangerous? [n (%)]		Extremely dangerous [118 (12.6)]	Dangerous [604 (64.7)]	Somewhat dangerous [202 (21.6)]	Not dangerous [10 (1.1)]^j^			
	Tablets	4.39 (1.75)	4.12 (1.40)	4.35 (2.09)	5.40 (1.96)	2.080	-	-
	Types of medications	4.00 (1.32)	3.97 (1.24)	4.04 (1.26)	4.60 (0.97)	0.258	-	-
Do you have an aversion to taking medicine? [n (%)]		Extremely dislike [25 (2.7)]	Dislike [287 (30.7)]	Mildly dislike [431 (46.1)]	No dislike [191 (20.4)]			
	Tablets	4.20 (2.75)	3.90 (1.61)^c, d^	4.23 (1.45)^c, e^	4.67 (1.77)^d, e^	7.859***	*f* = 0.17	1
	Types of medications	3.56 (1.08)^f^	3.68 (1.12)^g, h^	4.06 (1.29)^g, i^	4.39 (1.24)^f, h, i^	14.373***	*f* = 0.22	1

*p < 0.05, **p < 0.01, ***p < 0.001; ^a-i^ indicates significant differences between parameters (*p* < 0.05)

^a^ *d* = 0.18, 1-β = 0.65; ^b^ *d* = 0.55, 1-β = 0.77; ^c^ *d* = 0.22, 1-β = 0.82; ^d^ *d* = 0.46, 1-β = 1.00; ^e^ *d* = 0.28, 1-β = 0.90; ^f^ *d* = 0.68, 1-β = 0.89; ^g^ *d* = 0.31, 1-β = 0.98; ^h^ *d* = 0.61, 1-β = 1.00; ^i^ *d* = 0.26, 1-β = 0.85 (*d*: effect size for each post-hoc analysis, 1-β: observed power)

^j^excluded from analysis

**Table 5:** Perceptions of taking medications and ‘excessive threshold’

		Mean (SD)		*t* value/*F* value	Effect size	Observed power (1-β)
Experience with family members/close acquaintances who take “too many” medications [n (%)]		Yes [412 (44.1)]	No [522 (55.9)]			
	Tablets	4.00 (1.41)	4.38 (1.77)	3.652***	*d* = 0.23	0.94
	Types of medications	3.95 (1.24)	4.04 (1.26)	1.091	-	-

***p < 0.001

### Procedure

This questionnaire survey was conducted between April 2011 and December 2011. Students completed an anonymous, self-administered questionnaire survey regarding their perception of medications. We provided an oral explanation of the study, and considered their submission of the questionnaire survey as consent to participate in the study.

The protocol of this study was reviewed by the Secretarial Division of the Research Ethics Committee of the Kitasato University Kitasato Institute Hospital, and judged to require no further discussion by the Research Ethics Committee.

### Data and statistical analyses

First, we calculated the overall mean values and standard deviation for excessive thresholds for both tablets and types of medications taken in one sitting. We then summed the number of responses pertaining to each questionnaire item. Following this, to reveal whether questionnaire items affected the excessive threshold, we performed either a *t*-test or one-way analysis of variance (ANOVA) on the excessive threshold for both tablets and types of medications. For these tests, we excluded from the analysis any option selected by fewer than 20 participants. Any significant results obtained from the one-way ANOVA were subjected to post-hoc tests (Tukey or Games-Howell). Finally, in order to examine the multivariate association which accounted for the responses to each item concerning the excessive threshold for medications, we conducted multiple regression analysis (stepwise method, *p*_in_ < 0.05, *p*_out_ > 0.10) with each of the questionnaire items set as the explanatory variable, and excessive thresholds for both tablets and types of medications set as dependent variables. In that context, we realized that two questions (“Do you take medications as instructed (in the dosages prescribed) by the hospital?” and “If prescribed what you feel to be an excessive amount, do you still take the medications as instructed by the hospital?”) actually combined the queries of “whether or not medications are taken as instructed” and “forgetting to take medications” into one questionnaire item. As we felt that treating these two queries as one explanatory variable was inappropriate, we removed them from the explanatory variables. We calculated effect size and observed power for any variables found to be statistically significant by any given analysis.

The present study was exploratory, and data were analyzed using several statistical tools. While we found several statistically significant results with regard to the association with excessive thresholds for medications, some factors showed low power and thus require further examination. According to Cohen, an observed power of 0.80 or lower can create a high risk for type 2 statistical errors [7]. For results with a statistical power of 0.8 or higher, with the exception of the sense of trust in medications, univariate and multivariate analyses yielded similar results. Results with statistical power of 0.8 or higher will be addressed in the discussion section.

Statistical analyses were conducted using SPSS ver. 19 and G*Power 3, with statistical significance set at *p* < 0.05.

## Results

We received responses from a total of 1120 students. Of these, 163, 251, and 239 were 2nd year, 4th year, and 6th year pharmacy students, respectively, while 467 were students in non-medical schools. Questionnaire recovery rate was 81.4% among the pharmacy students. We excluded data from 186 students who submitted incomplete questionnaire surveys. Data from the remaining 934 students (333 males, 601 females; mean age, 20.6 ± 1.9 years; age range, 18-26 years) were included in the analysis.

### Excessive thresholds for medications overall

Mean values ± standard deviation for excessive thresholds for tablets and types of medications reported by all subjects were 4.21 ± 1.63 tablets and 4.00 ± 1.25 medications, respectively. The number of responses (%) was also summed for each item (Tables 2, 3, 4 and 5).

### Association between participant background and excessive threshold for medications

T-tests revealed that gender, university, and past medical history were all significantly associated with the excessive threshold for medications. Specifically, female participants, students in non-medical schools, and those who lacked past experience with major illnesses reached an excessive threshold with fewer tablets and types of medications compared to their counterparts with this experience (*t*(933) > 2.744, *p* < 0.01) (Table 2).

### Association between items related to taking medications and the excessive threshold for medications

The results of univariate analyses are shown in Table 3. Whether or not a participant was taking prescription medications was a significant factor (*t*(933) >2.198, *p* < 0.05), in that those who were not taking daily medications reached an excessive threshold with fewer tablets and fewer types of medications (Table 3). Supplement use was also a significant factor (*F*(2, 931) > 3.615, *p* < 0.05), and the post-hoc test showed that those who took supplements only when they were feeling unwell and those who did not take any supplements reached an excessive threshold with fewer tablets compared to those who took supplements on a daily basis. Those who did not take supplements reached an excessive threshold with fewer types of medications than those who took supplements on a daily basis.

### Association between the perception of medications and excessive threshold for medications

Results of the ANOVA are displayed in Table 4. Changes in the sense of trust and sense of aversion had a significant effect on the excessive threshold for medications (*F*(2, 922) > 4.070, *p* < 0.05; and *F*(3, 930) > 7.859, *p* < 0.01, respectively). The post-hoc test showed that those who feel that medications are reliable as well as those who feel that medications are somewhat reliable both reached an excessive threshold with fewer types of medications relative to those who reported that medications were extremely reliable (Table 4). The post-hoc tests did not find any significant differences with regard to the number of tablets.

With regard to the sense of aversion towards drugs, the post-hoc test found that those who reported extreme dislike of taking medications reached an excessive threshold with fewer types of medications compared to those who did not dislike taking medications. In addition, those who disliked taking medications reached an excessive threshold with significantly fewer tablets and types of medications compared to those who reported either mild or no dislike for taking medications. In addition, those who mildly disliked taking medications reached an excessive threshold with significantly fewer tablets and types of medications relative to those with no dislike for taking medications (Table 4).

### Association between attitudes and perceptions toward taking medications and the excessive threshold for medications

T-test and ANOVA results are displayed in Table 5 (results that were statistically significant and had a statistical power of 0.8 or higher) and Additional file 1 (all others). Those with experience with feeling that someone near to them was taking an excessive amount of drugs were significantly more likely to reach a consideration of ‘excessive’ with fewer tablets (*t*(933) = 3.652, *p* < 0.001). In addition, differences in the sense of displeasure when being prescribed what participants considered an excessive amount of medications affected the excessive threshold for medications (*F*(2, 930) > 4.477, *p* < 0.05). Post-hoc analysis showed that those who experienced a sense of displeasure when prescribed what they felt was an excessive amount of medication reached an excessive threshold with significantly fewer tablets and types of medications relative to those who did not feel displeasure. Differences in the understanding of drug efficacy when taking medications were also associated with the excessive threshold for number of tablets (*F*(3, 930) = 2.720, *p* < 0.05), but post-hoc test results were not significant.

### Factors that influence the excessive threshold for medications

**Fig. 1:**
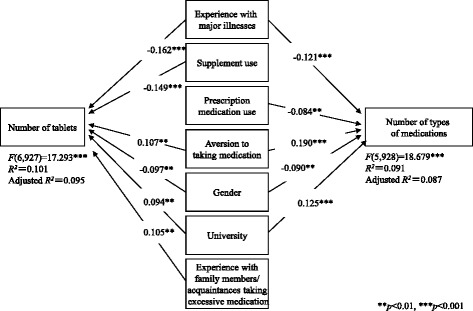
Association between factors that affected attitudes toward excessive threshold. Dummy variables. Experience with major illnesses: Yes = 1, No = 2; Supplement use: Yes = 1, Only when ill = 2, No = 3; Prescription medication use: Yes = 1, No = 2; Aversion to taking medication: Extremely dislike = 1, Dislike = 2, Mildly dislike = 3, No dislike = 4; Gender: Male = 1, Female = 2; University: Non-medical = 1, Pharmacy = 2; Experience with family members/acquaintances taking excessive medication: Yes = 1, No = 2

In order to examine multivariate associations between responses to each of the items in Tables 2, 3, 4 and 5 and excessive thresholds, we conducted multiple regression analyses. These revealed significant standardized regression coefficients for the association between the excessive threshold for types of medications and the five factors (Fig. 1), and between the excessive threshold for number of tablets and the six factors (Fig. 1), with no multicollinearity.

## Discussion

The present study examined factors associated with the amount of medications (numbers of tablets and types of medications) considered excessive for self-use. In particular, we focused on factors such as individual background variables, perceptions of medications, and attitudes toward taking medications. The present study revealed that those who dislike taking medications, those without prior experience with major illnesses, students in non-medical schools, females, and those who are not currently taking prescribed medications reached an excessive threshold with the lowest number of medication types. Furthermore, those with no experience with major illnesses, those who do not take supplements, those who dislike taking medicine, those who have felt that family or acquaintances were taking an excessive amount of medications, females, and students in non-medical schools reached an excessive threshold with the fewest number of tablets.

### Factors influencing attitudes toward medication dosage

Relative to males, females reached an excessive threshold with fewer tablets and types of medications. A previous survey on health awareness and lifestyles among university students found that females are comparatively more conscientious of their future health compared to males, and are also more anxious about their lifestyles [8]. As such, females may be more sensitive about putting foreign items such as drugs into their bodies, and this may explain why they exhibited excessive thresholds with fewer medications. Additionally, given that 1) more females request support for health consultations [8], 2) females between the ages of 13-44 years are prescribed more drugs [9], and, while the present study did not report this, our survey revealed that 3) among university students, female students reported that they ‘took medicine immediately’ more frequently than male students if they felt unwell, an extra layer of consideration may be required to support medication use for females. Students in non-medical schools reached an excessive threshold with fewer drugs than did pharmacy students. Pharmacy students who participated in the present study were 2nd, 4th, and 6th year students. Their perception of medications may have changed over the course of their education, or they simply may have possessed a different perspective on medication when entering the department. Pharmacy students are more constantly exposed to medications, and thus exhibit higher familiarity with them relative to students in non-medical schools, which may explain why students in non-medical schools had an excessive threshold with lower amounts of medications. Previous studies have found that, relative to medication specialists (pharmacists, prescriptionists), patients tend to think that medicine is a harmful substance [10]. We assume that this sentiment would be less pronounced among pharmacy students who hope to become future pharmacists.

Compared to those who experienced major illnesses, individuals without this experience reached an excessive threshold with fewer tablets and types of medications. In general, those who experience major illnesses take medications consistently for long periods. As this stimulus is repeated over and over again, the response elicited by the stimulus becomes weaker with time (a phenomenon known as “habituation”) [11], and is likely to occur with taking medications as well. Moreover, those who did not take daily supplements or who only took them when they felt unwell reached an excessive threshold with fewer tablets, relative to those who take them daily. Many illnesses elicit the prescription of several types of medications, but it is generally assumed that for each type, one tablet is taken—i.e., every time you take a medication, you will likely have to take multiple types of medication, and thus the concept of ‘excess’ is measured by the number of types of medications. In contrast, for supplements, which often require multiple tablets in one sitting, the concept of ‘excess’ is measured by the number of tablets taken in one sitting. These results demonstrate how habituation for taking prescription medications is based on the number of types of medications, while that for taking supplements is based on the number of tablets.

Individuals who had experienced a family member or close acquaintance take what they considered excessive amounts of medications reached an excessive threshold with fewer tablets relative to those without that experience. If an individual has a lower excessive threshold, then they may be sensitive to the amounts of medications taken by those around them. In fact, given the significant association between this experience and the excessive threshold for number of tablets, but not for types of medications, we speculate that their responses are based on visual judgments upon seeing the number of tablets.

Participants with a stronger dislike of medications exhibited lower excessive thresholds for medication amounts. This result is easy to comprehend. However, considering this from the perspective of Rozin’s theory on the sense of dislike [12], it is possible that the dislike of medications is somehow linked with the core displeasure comprising a combination of displeasure in the sense of wanting to protect oneself from poisonous/harmful substances, and the displeasure of swallowing such substances. Healthcare professionals should be aware of underlying reasons for any aversions causing patients to refuse medication. Reliability of drugs was also significantly associated with excessive thresholds. According to a survey from health insurance pharmacies, some individuals reported that “I would like to avoid taking medications as much as possible” or “the fewer medications to bring about healing the better”, while still others felt that “I am well because I am taking medications.” The latter group of individuals comprised a larger proportion of those with a high excessive thresholds (statistical significance unknown) [13]. Another survey that targeted pharmacology specialists (pharmacists, dispensing pharmacists, pharmacy technicians) found that those currently taking prescription drugs reported higher benefits of medications, relative to that reported by those who were not taking prescription drugs [14]. Individuals who experienced the effects of medications by taking them would be more apt to consider them reliable or beneficial. In addition, as mentioned above, the experience of taking medications also induces habituation toward medication amounts, which may help explain why these individuals reported a higher excessive threshold for medication amounts.

Multivariate regression analysis revealed that those who dislike taking medications, those without experience with major illnesses, students in non-medical schools, and females reached an excessive threshold with fewer tablets and types of medications. In addition, those who were not taking prescribed medications also reached an excessive threshold with fewer types of medications, while those who were not taking supplements and those who had experienced a family member or close acquaintance taking what they considered an excessive amount of medication reached an excessive threshold with fewer tablets. As demonstrated in the multivariate regression analysis, an individual’s perception of excess medication is associated with a variety of factors, and instructions for prescription medications should take into account differences in individual attitudes toward lifestyle conditions and medications, past experiences, and gender.

The present study targeted pharmacy students and students in non-medical schools. Other studies have reported variation in the perception of medications between specialists in pharmacology and the general populace [10], and a similar survey that compares pharmacists and the layperson should be conducted in Japan as well, to clarify differences in perception. Even among healthcare professionals, pharmacists, physicians, and nurses vary in their perception of medications [14, 15]. In order to provide appropriate guidance to patients for taking medications, a more detailed examination of differences in perception among healthcare professionals is needed.

### Limitations and strengths

Interpretation of the results of this study requires some caution. First, we did not examine age as an explanatory variable to determine the excessive thresholds for medications, primarily because all of our study participants were university students in the same age group. Furthermore, participants were limited to university students. However, previous studies have found that, relative to those in their 50s and above, younger individuals reach an excessive threshold with fewer types of medications [13], and so an analysis which incorporates age differences is warranted. Second, ‘experience with major illnesses’ was self-reported by participants, and was not measured objectively. Individual variation can occur in the perception of illnesses, and thus caution is required in the interpretation of results pertaining to this variable. Third, the multiple regression analysis produced a statistically significant model, but the coefficient of determination was small. Fourth, we did not thoroughly examine the differences between stated opinions on the number of tablets/types of medication considered excessive and the behavior of the individual toward taking their pills as prescribed. As the present study did not assess actual amounts of medication taken by participants, this will be an issue to address in future studies. Moreover, several variables deemed significant by our statistical tests showed low statistical power, suggesting the need for further examination.

In general, anyone can become a “patient” sometime in life. For instance, someone may become afflicted by a chronic disease at some point, and may require medication for prolonged periods. Many of the participants of this study were “young” and “healthy,” and our study was able to obtain information on their attitudes pertaining to starting a new medication.

### Improving medication adherence

The present study emphasized individual variation in perceptions surrounding medication, as well as the need for medication instructions to be tailored to each patient. The newly identified individual factors associated with amounts of medication considered excessive should be carefully considered when medication counseling is offered, as providing individualized counseling for addressable issues may potentially improve medication adherence. For instance, when misunderstandings about a particular drug arise due to differences in knowledge about medications (e.g., medical students vs. non-medical students), correcting the misunderstanding could lead to improved medication adherence. Medication guidance tailored to each individual will likely be necessary in the future.

### Future research

This study was in the piloting phase, and participants were limited to university students. Moreover, medication experience was based on self-reporting. Given the likely involvement of various background factors, the generalizability is limited. Although medication experience was one factor found to influence excessive thresholds for medication amounts, further investigation is required to examine indicators related to more regulated or objective medication experiences and how they influence excessive thresholds for medication amounts. In order to gain more insight into the awareness on taking medication, and to obtain further knowledge that could help increase medication adherence, a similar study targeting a more diverse study population is necessary. For instance, it would be helpful to increase participant age range, as well as conduct similar studies with a patient group but add a non-patient group as a control. Such studies have the possibility of providing new data that could further our understanding. Prospective studies targeting the present study population are also warranted.

## Conclusions

The present study noted gender-dependent variation in the perception of medication amounts, and identified several factors that affect excessive thresholds for medication amounts. Individual variation in the perception of medications exists, and thus medication instruction should be tailored to each patient. Further studies on these topics will be needed to facilitate a better understanding of patient attitudes toward medications and construct better relationships between patients and their pharmacists.

## Additional file

Additional file 1: Attitudes and perceptions of taking medications and ‘excessive threshold’. This table shows the results of associations between attitudes and perceptions toward taking medications and the excessive threshold for medications, excluding those with statistical significance and a statistical power of 0.8 or higher. (PPTX 47 kb)

## Abbreviation

ANOVA: Analysis of variance
