# Supplementary material for: Factors associated with medication amounts considered excessive among university students: a questionnaire survey of pharmacy students and those in non-medical schools
Source: BMC Health Serv Res. 2017 Jul 11;17:475. doi: 10.1186/s12913-017-2431-9 (PMC5505140; doi:10.1186/s12913-017-2431-9)
Supplement: Additional file 1: — Attitudes and perceptions of taking medications and ‘excessive threshold’. This table shows the results of associations between attitudes and perceptions toward taking medications and the excessive threshold for medications, excluding those with statistical significance and a statistical power of 0.8 or higher. (PPTX 47 kb) [file 12913_2017_2431_MOESM1_ESM.pptx]

## Slide 1
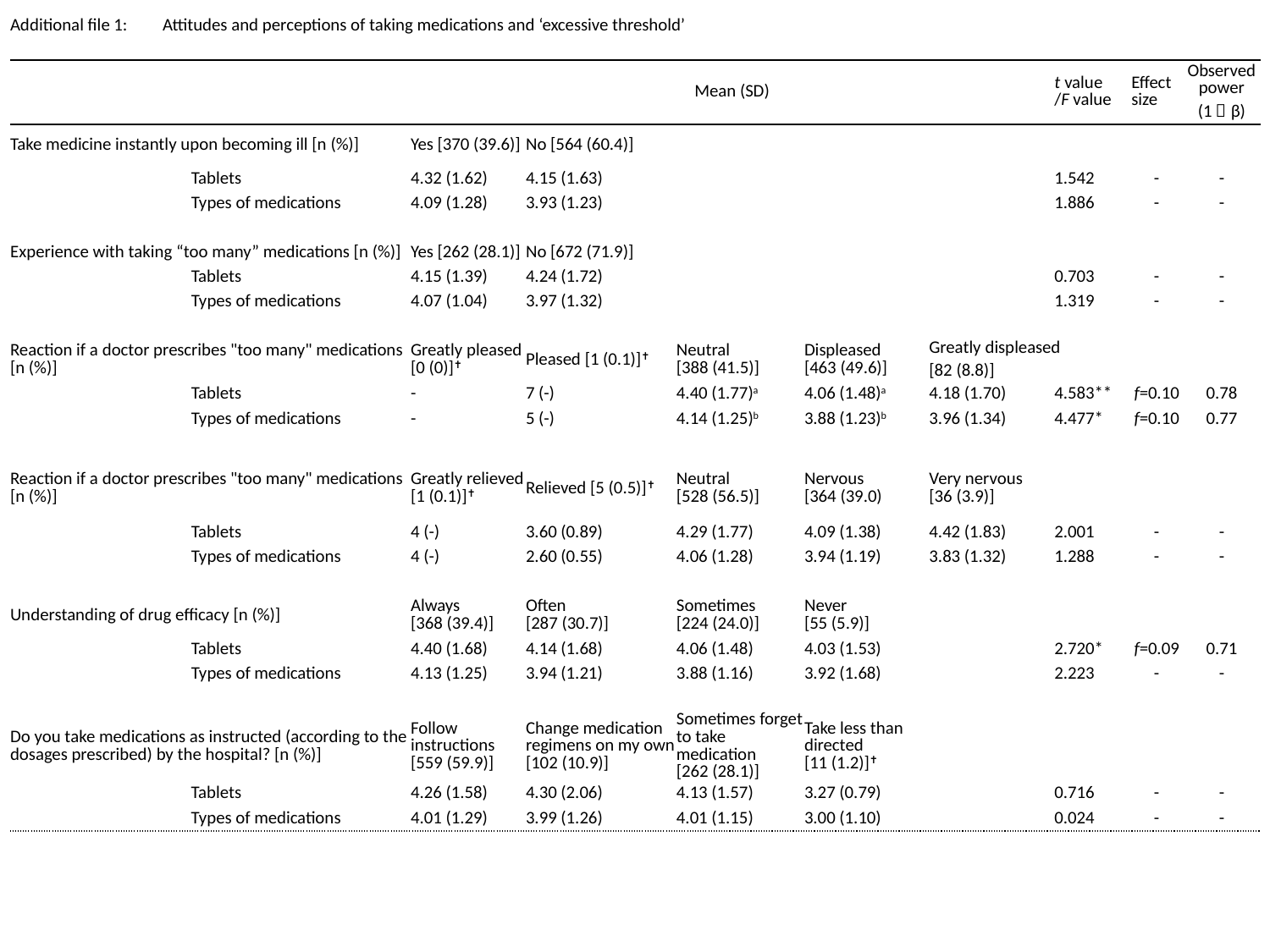

| Additional file 1:　 Attitudes and perceptions of taking medications and ‘excessive threshold’ | | | | | | | | | | |
| --- | --- | --- | --- | --- | --- | --- | --- | --- | --- | --- |
| | | | | | | | | | | |
| | | | Mean (SD) | | | | | t value /F value | Effect size | Observed power (1－β) |
| Take medicine instantly upon becoming ill [n (%)] | | | Yes [370 (39.6)] | No [564 (60.4)] | | | | | | |
| | Tablets | | 4.32 (1.62) | 4.15 (1.63) | | | | 1.542 | - | - |
| | Types of medications | | 4.09 (1.28) | 3.93 (1.23) | | | | 1.886 | - | - |
| | | | | | | | | | | |
| Experience with taking “too many” medications [n (%)] | | | Yes [262 (28.1)] | No [672 (71.9)] | | | | | | |
| | Tablets | | 4.15 (1.39) | 4.24 (1.72) | | | | 0.703 | - | - |
| | Types of medications | | 4.07 (1.04) | 3.97 (1.32) | | | | 1.319 | - | - |
| | | | | | | | | | | |
| Reaction if a doctor prescribes "too many" medications [n (%)] | | | Greatly pleased [0 (0)]✝ | Pleased [1 (0.1)]✝ | Neutral [388 (41.5)] | Displeased [463 (49.6)] | Greatly displeased [82 (8.8)] | | | |
| | Tablets | | - | 7 (-) | 4.40 (1.77)a | 4.06 (1.48)a | 4.18 (1.70) | 4.583\*\* | f=0.10 | 0.78 |
| | Types of medications | | - | 5 (-) | 4.14 (1.25)b | 3.88 (1.23)b | 3.96 (1.34) | 4.477\* | f=0.10 | 0.77 |
| | | | | | | | | | | |
| Reaction if a doctor prescribes "too many" medications [n (%)] | | | Greatly relieved [1 (0.1)]✝ | Relieved [5 (0.5)]✝ | Neutral [528 (56.5)] | Nervous [364 (39.0) | Very nervous [36 (3.9)] | | | |
| | Tablets | | 4 (-) | 3.60 (0.89) | 4.29 (1.77) | 4.09 (1.38) | 4.42 (1.83) | 2.001 | - | - |
| | Types of medications | | 4 (-) | 2.60 (0.55) | 4.06 (1.28) | 3.94 (1.19) | 3.83 (1.32) | 1.288 | - | - |
| | | | | | | | | | | |
| Understanding of drug efficacy [n (%)] | | | Always [368 (39.4)] | Often [287 (30.7)] | Sometimes [224 (24.0)] | Never [55 (5.9)] | | | | |
| | Tablets | | 4.40 (1.68) | 4.14 (1.68) | 4.06 (1.48) | 4.03 (1.53) | | 2.720\* | f=0.09 | 0.71 |
| | Types of medications | | 4.13 (1.25) | 3.94 (1.21) | 3.88 (1.16) | 3.92 (1.68) | | 2.223 | - | - |
| | | | | | | | | | | |
| Do you take medications as instructed (according to the dosages prescribed) by the hospital? [n (%)] | | | Follow instructions [559 (59.9)] | Change medication regimens on my own [102 (10.9)] | Sometimes forget to take medication [262 (28.1)] | Take less than directed [11 (1.2)]✝ | | | | |
| | Tablets | | 4.26 (1.58) | 4.30 (2.06) | 4.13 (1.57) | 3.27 (0.79) | | 0.716 | - | - |
| | Types of medications | | 4.01 (1.29) | 3.99 (1.26) | 4.01 (1.15) | 3.00 (1.10) | | 0.024 | - | - |
| | | | | | | | | | | |

## Slide 2
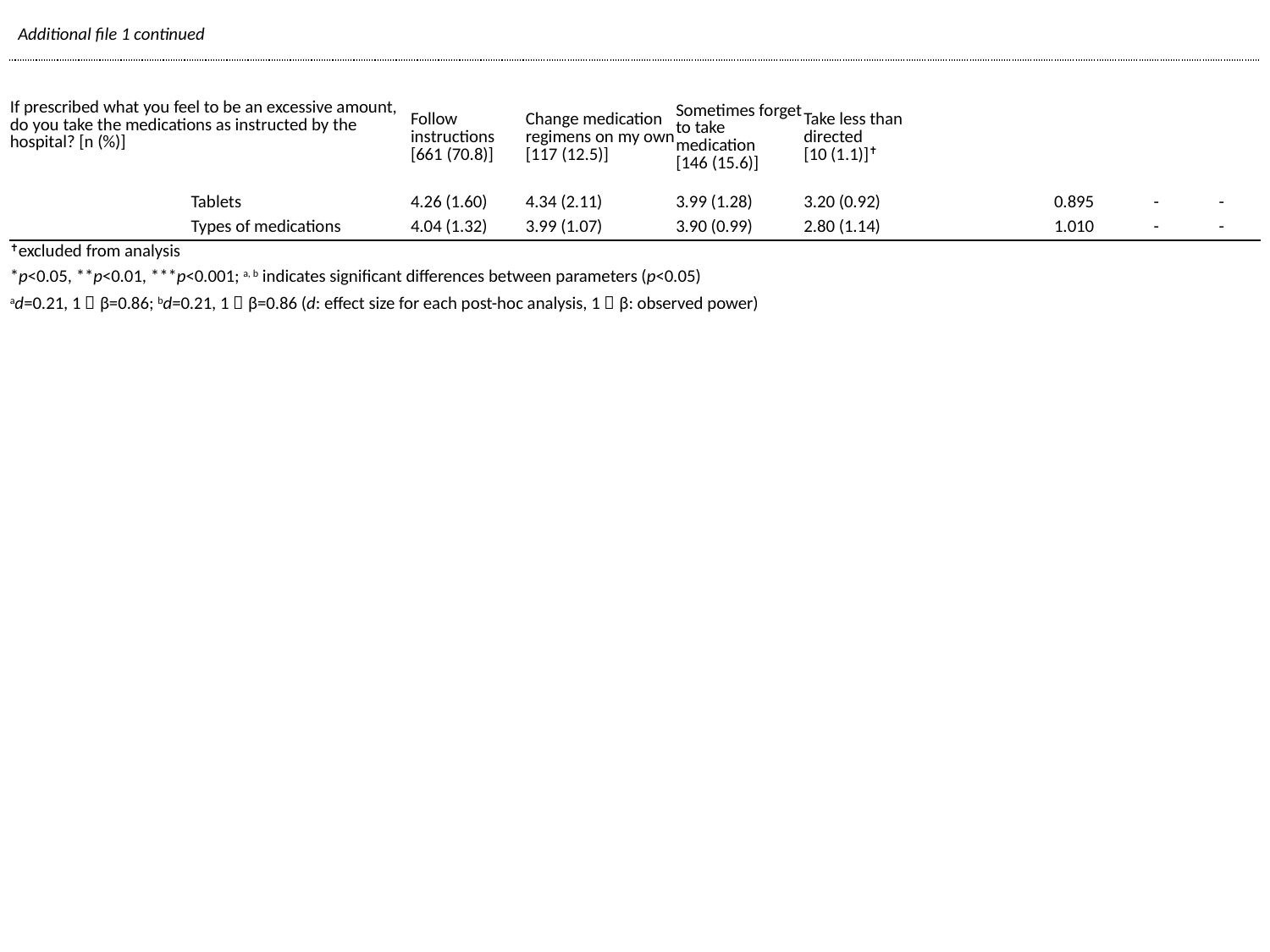

Additional file 1 continued
| | | | | | | | | | |
| --- | --- | --- | --- | --- | --- | --- | --- | --- | --- |
| If prescribed what you feel to be an excessive amount, do you take the medications as instructed by the hospital? [n (%)] | | Follow instructions [661 (70.8)] | Change medication regimens on my own [117 (12.5)] | Sometimes forget to take medication [146 (15.6)] | Take less than directed [10 (1.1)]✝ | | | | |
| | Tablets | 4.26 (1.60) | 4.34 (2.11) | 3.99 (1.28) | 3.20 (0.92) | | 0.895 | - | - |
| | Types of medications | 4.04 (1.32) | 3.99 (1.07) | 3.90 (0.99) | 2.80 (1.14) | | 1.010 | - | - |
| ✝excluded from analysis | | | | | | | | | |
| \*p<0.05, \*\*p<0.01, \*\*\*p<0.001; a, b indicates significant differences between parameters (p<0.05) | | | | | | | | | |
| ad=0.21, 1－β=0.86; bd=0.21, 1－β=0.86 (d: effect size for each post-hoc analysis, 1－β: observed power) | | | | | | | | | |
